# The complete chloroplast genome of *Persicaria maackiana* (Regel) Nakai ex T. Mori (Polygonaceae) in Korea

**DOI:** 10.1080/23802359.2022.2119821

**Published:** 2022-09-15

**Authors:** Kang-Rae Kim, So Young Park, Sun-Yu Kim, Young Taek Oh, Jeong-Nam Yu

**Affiliations:** Animal & Plant Research Department, Nakdonggang National Institute of Biological Resources, Sangju, Republic of Korea

**Keywords:** *Persicaria maackiana*, Polygonaceae, chloroplast genome, Illumina sequencing

## Abstract

*Persicaria maackiana* (Regel) Nakai ex T. Mori (1922), a species of the Polygonaceae family, is an annual plant widely distributed in Northeast Asia. We aimed to sequence the complete chloroplast genome of *P. maackiana* using Illumina HiSeq paired-end sequencing. The chloroplast genome was determined to be 160,635 bp. The complete chloroplast genome contained 129 genes, including 84 protein-coding genes, 37 tRNA, and eight rRNA genes. Phylogenetic analysis of the chloroplast genome sequences of 15 Polygonaceae plants revealed that *P. maackiana* was most closely related to *P. perfoliata*. Our findings might be useful for future phylogenetic studies of Polygonaceae.

*Persicaria maackiana* (Regel) Nakai ex T. Mori is an annual plant of the Polygonaceae family, and the genus *Persicaria*. *P. maackiana* inhabits lowlands with fresh water, and is widely distributed in Northeast Asia – from southeastern China to Japan, Siberia, and the Korean Peninsula (Ohwi 1965; Komarov et al. 1968; Li et al. 2003; Chang et al. 2014). Numerous plant species of the Polygonaceae family have been used medicinally since ancient times (Wang et al. 1988; Hussain et al. 2010; Khatun et al. 2015; Tonny et al. 2017). Particularly, plants belonging to the genus *Persicaria* have often been used as diuretics and anti-inflammatory as well as to treat skin diseases, such as ringworm and boils (Khatun et al. 2015; Tonny et al. 2017). Recently, *P. maackiana* extract has been studied for its antidiabetic effect by promoting glucose absorption in human cells, and has been identified as a potential medicinal plant in Korea (NNIBR 2021). Despite its potential economic value, molecular genetic studies of *P. maackiana* have not been conducted in Korea. Therefore, we sequenced the complete chloroplast genome of *P. maackiana* as a first step to elucidate its genetic characteristics.

The *P. maackiana* leaf samples used in this study were collected from the Gaecheon Reservoir (36°23′42″ N, 128°27′56″ E) in Uiseong-gun, Gyeongsangbuk-do, South Korea (storage: Nakdonggang National Institute of Biological Resources; voucher number: NNIBRVP70284, email: ksuny007@nnibr.re.kr). High-quality genomic DNA was extracted using the DNeasy^®^ Plant Mini Kit (Qiagen, Hilden, Germany) following the manufacturer’s protocol. Genomic DNA was sequenced using Illumina HiSeq sequencing with a 150 bp paired-end library. Moreover, 145 Gb of raw reads were obtained using Illumina HiSeq 2500 sequencing. We used NOVOPlasty v.4.3.1 (Dierckxsens et al. 2017) to assemble the complete chloroplast genome and CPGAVAS2 (Shi et al. 2019) to annotate the genome (Kearse et al. 2012). In addition, erroneous annotations were checked using National Center for Biotechnology Information (NCBI) BLAST and manually corrected using Geneious 11.0.12 software. The MISA tool (http://pgrc.ipk-gatersleben.de/misa/misa.html) was used to identify simple sequence repeat (SSR) regions in the chloroplast genome. The annotated chloroplast genome of *P. maackiana* was deposited into GenBank with the accession number OM386813.

The length of the complete chloroplast genome of *P. maackiana* was 160,635 bp, and the GC content was 37.9%. The GC content of the chloroplast genome of the genus *Persicaria* was 37.8%, 38.0%, and 38.0% in *P. filiformis*, *P. chinensis*, and *P. perfoliata*, respectively. Notably, the GC content of *P. perfoliata* chloroplast genome was most similar to that of *P. maackiana*.

The chloroplast genome of *P. maackiana* had a quadrilateral structure with a large single-copy (LSC) region of 85,375 bp, a short single-copy (SSC) region of 13,095 bp, and two inverted repeat (IR) regions of 31,131 bp. Furthermore, 129 functional genes were encoded, which included 84 protein-coding, 37 tRNA, and eight rRNA genes. We identified 51 SSRs, including 32 mononucleotides, six di-nucleotides, five tri- and tetra-nucleotides, two penta-nucleotides, and one hexa-nucleotide (S1 Table). Most SSRs were identified in the LSC and SSC regions (41), and the remaining in IR region a (5) and IR region b (5).

To determine the phylogenetic location of *P. maackiana*, chloroplast genome sequence of 15 Polygonaceae species was aligned using the MAFFT v.7.490 automated algorithm (Katoh and Standley 2013). The optimal GTR + G + I model was applied according to the Akaike information criterion using jModelTest v.2.1.7 to obtain the optimal model sequence (Posada 2008). The phylogenetic tree was reconstructed using the PhyML 3.0 program as the maximum-likelihood (ML) method, and 100 bootstrap replicates were performed (Guindon et al. 2010). Phylogenetic trees were visualized and manually edited using Figtree v1.4.4 (Rambaut 2018).

The phylogenetic tree was divided into two clades, Persicarieae and Fagopyreae tribes, with Fagopyreae as an external group. The first clade, Persicarieae, was clustered with the species of the genera *Persicaria* and *Bistorta*, and the species of the genus *Persicaria* formed a single clade with very high bootstrap values of over 99% (Figure 1). The phylogenetic results of the ML analysis revealed that *P. maackiana* was most closely related to *P. perfoliata*. Notably, previous classical classification and phylogenetic results indicate that *P. maackiana* clusters with species of the section Echinocaulon (Park 1986; Kim and Donoghue 2008; Schuster et al. 2015). In this study, *P. maackiana* was clustered with *P. perfoliata* of the section Echinocaulon, which is consistent with previous studies (Park 1986; Kim and Donoghue 2008; Schuster et al. 2015). The *P. maackiana* chloroplast genome we sequenced might provide a solid basis for future genome-based phylogenetic and evolutionary relationship studies of Polygonaceae.

**Figure 1.**
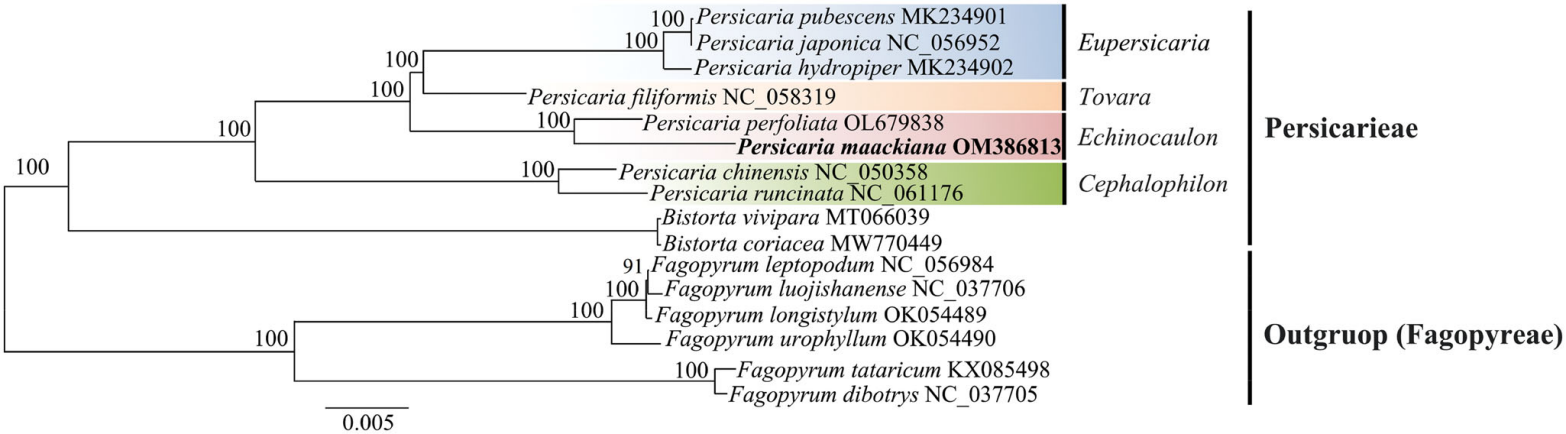
A phylogenetic tree was constructed using the maximum-likelihood method based on complete chloroplast sequence from 16 species. The numbers above the nodes represent the bootstrap support values for each branch.

## Ethical approval

No permission from the Republic of Korea government was required to collect these plants.

## Consent form

The authors complied with relevant institutional (Nakdonggang National Institute of Biological Resources), national (Republic of Korea), and international guidelines (IUCN) and legislation for the plant study.

## Author contributions

Kang-Rae Kim: conceptualization and data curation, NGS data analysis, writing – original draft, writing – review, and editing. So Young Park: NGS data analysis and data curation. Sun-Yu Kim: conceptualization, sampling, and investigation. Young Taek Oh: conceptualization, sampling, and investigation. Jeong-Nam Yu: conceptualization, data curation, supervision, funding acquisition, project administration, writing – review, and editing.

## Supplementary Material

Supplemental Material

## Data Availability

The genome sequence data that support the findings of this study are openly available in GenBank of NCBI at https://www.ncbi.nlm.nih.gov/ under the accession number OM386813. The associated BioProject, SRA, and Bio-Sample numbers are PRJNA826290, SRR18740272, and SAMN27554296, respectively.
